# High-Resolution Melting (HRM) Curve Assay for the Identification of Eight *Fusarium* Species Causing Ear Rot in Maize

**DOI:** 10.3390/pathogens9040270

**Published:** 2020-04-07

**Authors:** Simon Schiwek, Lukas Beule, Maria Vinas, Annette Pfordt, Andreas von Tiedemann, Petr Karlovsky

**Affiliations:** 1 Molecular Phytopathology and Mycotoxin Research, University of Goettingen, 37077 Goettingen, Germany; lukas.beule@agr.uni-goettingen.de; 2 Centro para Investigaciones en Granos y Semillas, University of Costa Rica, 2060 San Jose, Costa Rica; maria.vinasmeneses@ucr.ac.cr; 3 Plant Pathology and Crop Protection, University of Goettingen, 37077 Goettingen, Germany; annette.pfordt@uni-goettingen.de (A.P.); atiedem@gwdg.de (A.v.T.)

**Keywords:** *Fusarium*, high-resolution melting (HRM) curves, HRM analysis, maize ear rot, fungal colony PCR, *RPB2*, *TEF-1α*

## Abstract

Maize plants are often infected with fungal pathogens of the genus *Fusarium*. Taxonomic characterization of these species by microscopic examination of pure cultures or assignment to mating populations is time-consuming and requires specific expertise. Reliable taxonomic assignment may be strengthened by the analysis of DNA sequences. Species-specific PCR assays are available for most *Fusarium* pathogens, but the number of species that infect maize increases the labor and costs required for analysis. In this work, a diagnostic assay for major *Fusarium* pathogens of maize based on the analysis of melting curves of PCR amplicons was established. Short segments of genes *RPB2* and *TEF-1α*, which have been widely used in molecular taxonomy of *Fusarium*, were amplified with universal primers in a real-time thermocycler and high-resolution melting (HRM) curves of the products were recorded. Among major *Fusarium* pathogens of maize ears, *F. cerealis, F. culmorum, F. graminearum, F. equiseti, F. poae, F. temperatum, F. tricinctum*, and *F. verticillioides*, all species except for the pair *F. culmorum/F. graminearum* could be distinguished by HRM analysis of a 304 bp segment of the *RPB2* gene. The latter two species could be differentiated by HRM analysis of a 247 bp segment of the *TEF-1α* gene. The assay was validated with DNA extracted from pure cultures of fungal strains, successfully applied to total DNA extracted from infected maize ears and also to fungal mycelium that was added directly to the PCR master mix (“colony PCR”). HRM analysis thus offers a cost-efficient method suitable for the diagnosis of multiple fungal pathogens.

## 1. Introduction

Infection of crop plants with *Fusarium* spp. causes yield losses and leads to contamination of grains with mycotoxins [1]. *Fusarium* ear rot and ear mold are cosmopolitan diseases of maize, caused by *Fusarium* species producing secondary metabolites toxic to mammals, which are called mycotoxins. The most important mycotoxins found in maize grains are trichothecenes, zearalenone, and fumonisins. These contaminants impair grain quality and pose a risk to food safety [2,3]. Pre-harvest ear rot disease of maize is characterized by the appearance of white or reddish fungal mycelium with rotting symptoms on the cob. The disease is classified into two groups: *Gibberella* ear rot, also known as red ear rot, which is caused predominantly by *F. graminearum*, and *Fusarium* ear rot, also known as *Fusarium* ear mold and pink ear rot, which is caused by *F. verticillioides.* The most important infection route of maize cobs for both pathogens is colonization of silks [4,5], while *F. verticillioides* can also systemically colonize plants [6,7,8,9]. Other *Fusarium* species such as *F. temperatum*, *F. subglutinans*, *F. poae*, *F. cerealis*, *F. tricinctum*, and *F. culmorum* have also been reported to infect maize plants [10,11,12,13,14,15]. In the past decade, mycotoxins primarily known from maize and their producers have been reported also in other crops. The infection of wheat [16,17] and asparagus [18] with fumonisin-producing species of the *Gibberella fujikuroi* species complex is well established. Other species, not previously known to be infected by *F. verticillioides* in the field, such as rice and sugar beet, were shown to be susceptible to the pathogen when artificially infected [19]. In addition, weeds in maize fields were found to be heavily colonized by *Fusarium* spp., which are pathogenic on maize [20]. Residues of these plants might be the source of infection of maize in the next season. Infestation of the ears [21] and roots [22] of maize by herbivores facilitates infection by breaking mechanical barriers and disseminating inoculum. Due to the multiplicity of sources of inoculum and the complexity of factors affecting infection, the contamination of maize with *Fusarium* toxins is highly variable and difficult to predict [11,14].

Identification of *Fusarium* species can be achieved via a combination of phenotypic characterization (micro-/macromorphology) [23], assignment to mating populations [24,25], and analysis of selected gene loci [26,27,28]. Furthermore, production of specific secondary metabolites can support taxonomic assignments of *Fusarium* spp. [29,30,31]. Phenotypic traits alone are often not sufficient for taxonomical classification at the species level, especially regarding members of species complexes such as the *F. fujikuroi* species complex (FFSC) [13] or the *F. oxysporum* species complex (FOSC) [32]. Molecular tools are, therefore, widely used. Species-specific PCR primers [33,34,35] and real-time PCR assays [31,36,37,38,39] have been developed for all economically important *Fusarium* species. Previous studies have reported that sequencing of several marker genes, such as the RNA polymerase II second largest subunit (*RPB2*), translation elongation factor 1 alpha (*TEF-1α*), and beta-tubulin (*β-TUB*) [26,28,40,41], enables reliable distinction at the species level. However, only minor nucleotide variations or single nucleotide polymorphisms (SNPs) may distinguish between closely related species, as was observed for the differentiation of *F. temperatum* from *F. subglutinans* [13]. Therefore, for robust taxonomical classification, the use of additional marker genes is recommended.

Species-specific PCR assays are available for all economically relevant *Fusarium* species, but carrying out numerous assays for each sample multiplies the costs. Multiplexing reduces the costs of polymerase and nucleotides (not the costs of primers), but it adds the need to separate the signals. Electrophoretic separation of PCR products is not scalable; therefore, fluorescence-based species-specific detection is used, but minisequencing [27] and double-labeled hybridization probes [36] significantly increase the costs of such assays. 

Melting curve analysis is a closed-tube technique for the characterization of genetic variation in DNA amplicons based on the dissociation of double-stranded DNA with increasing temperature [42]. The amount of double-stranded DNA in each step is determined by the fluorescence of DNA-intercalating dye. High-resolution melting (HRM) curves generated with small temperature increments (commonly 0.1–0.2 °C) allow DNA fragments differing by as little as a single nucleotide to be distinguished. The entire analysis is carried out in the real-time PCR thermocycler that was used for the amplification. SYBR Green^®^ is the standard dye used in real-time PCR, but EvaGreen^®^ is used instead in melting curve analysis because it binds to all DNA base pairs [43]. Melting curves reflect not just GC composition, but also the sequence of the amplicon, and can therefore differentiate among amplicons with identical GC content. The analysis of DNA melting curves has successfully been applied in clinical medicine [44], virology [45], and in the identification of plants [46], insects [47], and phytopathogenic fungi [48,49].

In the present study, the suitability of melting curve analysis of short variable subsections of *RPB2* and *TEF-1α* genes for the differentiation of eight major *Fusarium* pathogens infecting maize ears in Germany [50] was established. 

## 2. Results

### 2.1. HRM Analysis of *sRPB2* and *sTEF-1α* for the Identification of *Fusarium* Species

**Figure 1:**
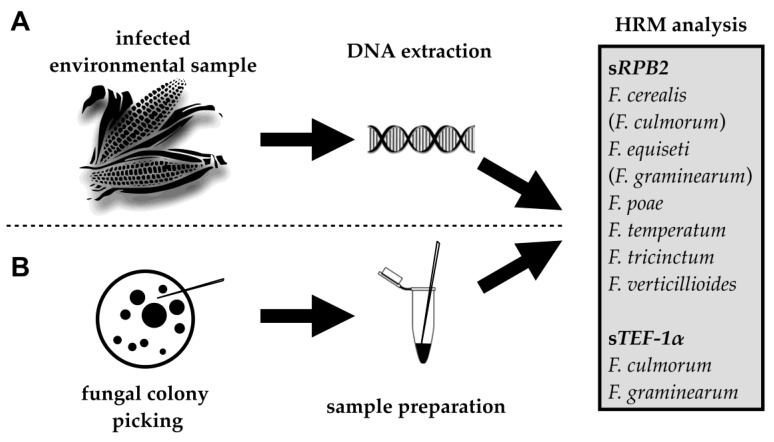
Workflow of high-resolution melting (HRM) curve analysis of eight major *Fusarium* pathogens of maize ears. (**A**) Identification of *Fusarium* species in infected maize ear samples; (**B**) identification or pure cultures using fungal colony PCR. s*RPB2* and s*TEF-1α* are short and variable subsections of *RPB2* and *TEF-1α*, suitable for HRM analysis (Table 1). *Fusarium* species in brackets were not well distinguishable using the s*RPB2* assay.

**Figure 2:**
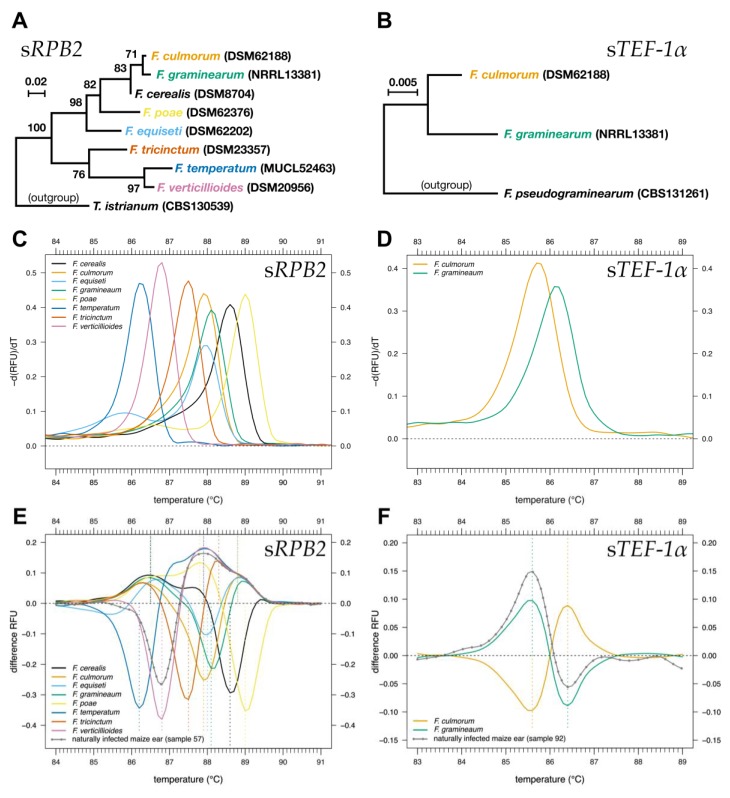
Maximum likelihood analysis of the DNA sequences used for HRM analysis. (**A**) s*RPB2* sequences (1000 bootstrap replications) (**B**) s*TEF-1α* (without bootstrapping due to a low number of sequences (*n* = 3)). We included partial sequences of *RPB2* of *Trichoderma istrianum* CBS130539 (Accession KJ665281.1) and *TEF-1α* of *F. pseudograminearum* CBS131261 (Accession JX118971.1) as outgroup references. Melting curves using normalized relative fluorescence unit (RFU) data of s*RPB2* (**C**) and s*TEF-1α* (**D**). Melting curves were generated as negative first derivative (−d(RFU)/d(T)) of relative fluorescence. Difference curves of the reference strains and a naturally infected maize ear for s*RPB2* (**E**) and s*TEF-1α* (**F**) are shown. The difference curves were obtained by subtracting melting curve data of each reference strain or environmental sample from the mean melting curves of all reference strains (dashed horizontal line). Vertical dashed lines indicate the maximum and minimum for each reference strain. s*RPB2* and s*TEF-1α* are short subsections of *RPB2* and *TEF-1α* (Table 1).

Primers commonly used for the amplification of *RPB2* and *TEF-1α* in taxonomy amplify fragments that are too long for HRM analysis. Therefore, new primers were developed for the identification of eight major *Fusarium* species that cause ear rot in maize, namely *F. cerealis, F. culmorum, F. graminearum, F. equiseti*, *F. poae*, *F. temperatum*, *F. tricinctum*, and *F. verticillioides*, via HRM analysis (Table 1, Figure S1). Taxonomically characterized reference strains were used as standards (Table S1). Short variable segments of *RPB2* and *TEF-1α* genes, which we refer to herein as s*RPB2* (short*RPB2,* 304 bp) and s*TEF-1α* (short*TEF-1α,* 247 bp), respectively (Table 1), were used. s*RPB2* reliably distinguished *F. cerealis, F. equiseti*, *F. poae*, *F. temperatum*, *F. tricinctum*, and *F. verticillioides* (Figure 1, Figure 2C,E). The melting curves allowed secure discrimination of DNA of these pathogens extracted from pure culture as well as from naturally infected maize ears. s*RPB2* amplicons of *F. culmorum* and *F. equiseti* had similar melting temperatures but could be distinguished with an additional melting domain of the amplicon of *F. equiseti*, which caused a shoulder in the melting curve (Figure 2C). The differentiation between *F. culmorum* and *F. graminearum* was more difficult, due to highly similar melting curves. However, the melting curves of the s*TEF-1α* fragment allowed reliable differentiation between *F. culmorum* and *F. graminearum* DNA extracted from pure cultures as well as from infected maize cobs (Figure 2D,F). Therefore, HRM for the s*TEF-1α* fragment was included in the assay. Both s*RPB2* and s*TEF-1α* were amplified using identical PCR conditions. The simultaneous amplification within the same PCR run enabled the identification of all eight tested *Fusarium* species in a single HRM analysis (Figure 2C–F). 

Melting temperatures of PCR products of the reference strains are listed in Table S2. The GC content ranged from 49 to 54% for s*RPB2* and 49 to 50% for s*TEF-1α* (Table S2, Figure S1). No primer dimers or unspecific products were observed for the reference strains. Nucleotide sequences obtained for both s*RPB2* and s*TEF-1α* showed high similarity (≥ 85%) across the reference strains (Figure 2A,B, Figure S1). In total, 73 SNPs were found in s*RPB2* and 4 SNPs in s*TEF-1α* (Figure S1). Nucleotide polymorphisms were relatively evenly distributed across the length of s*RPB2* (Figure S1A), but clustered in s*TEF-1α* (Figure S1B). Comparing the number of DNA polymorphisms with the melting curves, we concluded that the minimum number of nucleotide differences sufficient for differentiation between two *Fusarium* species by melting curve analysis was four. In the reference strains of *F. graminearum* and *F. culmorum*, these differences occurred at nucleotide positions 102, 158, 188, and 190 of s*TEF-1α* (Figure S1B). In s*RPB2*, only two distant nucleotide positions differed between the reference strains of these species (nucleotide positions 104 and 275). Based on s*RPB2*, no reliable separation of *F. graminearum* and *F. culmorum* by HRM was possible. A maximum number of 46 SNPs were observed in s*RPB2* sequences of the reference strains for *F. temperatum* and *F. poae* (Figure S1A).

The specificity of the assay was assessed by determining the melting temperatures of s*RPB2* and s*TEF-1α* amplicons for an additional 12 *Fusarium* species (Table S4). Except for four species, melting temperatures of s*RPB2* amplicons differed from the melting temperatures of s*RPB2* of all target species (Table S2) by more than 0.15 °C. Only *F. redolens*, *F. proliferatum*, *F. fujikuroi*, and *F. avenaceum* could not be differentiated from some of the target species by the melting temperature of s*RPB2*. The amplification of s*TEF-1α* failed for *F. redolens* and *F. avenaceum* (Table S4), distinguishing them from the target species. *F. proliferatum* and *F. fujikuroi* could not be distinguised from *F. temperatum* by melting temperatures of s*RPB2* or s*TEF-1α* amplicons; it has to be noted that *F. fujikuroi* does not infect maize. 

Both amplicons, used to generate melting curves, were generated with primer pairs consisting of a well-established primer (RPB2-5F2 and EF1αR) and a new primer designed for this study (RPB2-5R1s and TEF-1aFs2). The presence of binding sites for the established primers in all *Fusarium* spp. has been documented in numerous studies but the robustness of the newly designed primers was unknown. Nucleotide variation in binding sites might lead to a failure of the method with field isolates. To assess primer binding to DNA from other strains, 64 sequences of *RPB2* or *TEF-1α* from isolates of target species were retrieved and aligned with the sequences of newly designed primers. Not a single mismatch was found; the list of the sequences is provided in Table S5. Regarding *F. graminearum* Schwabe, many genetic lineages of this traditional species have been defined as species, although the boundaries of the new species are incongruent with the biological species concept and remain controversial [23]. To check for binding of newly designed primers to target sequences from these lineages, we retrieved 28 sequences of *RPB2* and *TEF-1α* from members of seven phylogenetic lineages of *F. graminearum* sensu lato. Aligning the sequences with primers RPB2-5R1s or TEF-1aFs2 did not reveal any mismatch (Table S6). 

### 2.2. Identification of *Fusarium* Species in Naturally Infected Maize Ears

The HRM assay was evaluated by screening DNA samples extracted from 100 maize ears naturally infected with *Fusarium* spp. (Figure 1A), which were sampled from across Germany (Figure S2). Morphological examination of fungal strains isolated from these ears prior to DNA extraction revealed that most ears were infected with several *Fusarium* species. HRM analysis successfully identified the most abundant *Fusarium* species in 80% of the ears (Table S3). The majority of maize ears (62%) were infected with *F. graminearum*, followed by *F. verticillioides* (10%), *F. temperatum* (6%), and *F. poae* (2%).

### 2.3. Fungal Colony PCR

**Figure 3:**
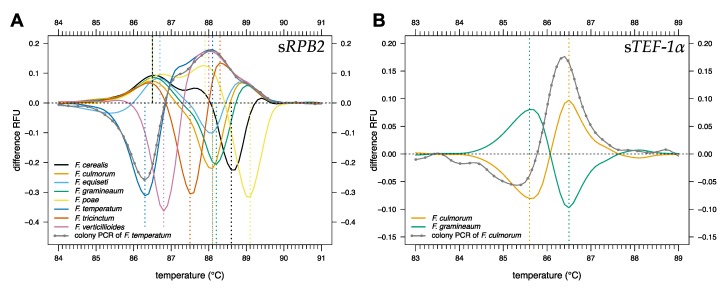
Fungal colony PCR followed by high-resolution melting (HRM) curve analysis of (**A**) s*RPB2* and (**B**) s*TEF-1α*. Difference curves were obtained by subtracting melting curves of each reference strain from the mean melting curve data of all reference strains (dashed horizontal line). Vertical dashed lines indicate the maximum and minimum of the curve for each reference strain. s*RPB2* and s*TEF-1α* are short subsections of *RPB2* and *TEF-1α* (Table 1).

All eight reference strains of *Fusarium* were successfully identified via HRM analysis after colony PCR (Figure 3). Boiling a small piece of mycelium picked from an agar plate for 10 min in 100 μL H_2_O released sufficient amounts of DNA for amplification. The use of larger amounts of mycelium for DNA preparation by boiling occasionally led to the inhibition of PCR; thus, a very small piece of mycelium (just visible by the naked eye) was sufficient. The inhibition of PCR by mycelial extracts was particularly pronounced for *F. poae*: Extracts of 100 µg mycelium (dry weight) boiled in 100 µL water always inhibited PCR, while extracts of 10 µg mycelium reliably generated the desired amplicons. 

## 3. Discussion

Analysis of melting curves of PCR products has been used previously to diagnose pathogens [51,52], including a duplex assay for two *Fusarium* species [37] and intraspecific differentiation within a *Fusarium* species complex [49]. In this work, the analysis of melting curves of PCR amplicons was exploited for the development of a multiplex diagnostic assay. *Fusarium* species commonly infecting maize ears in Germany [50] were selected for the implementation of the concept, but disease diagnosis in many crops faces the same challenge: numerous pathogens can infect the crop, though only a single pathogen or a few pathogens are typically found in each sample. For instance, many *Fusarium* pathogens can be isolated from ears of small grain cereals afflicted with *Fusarium* Head Blight [53]. To overcome the limit of multiplexing species-specific PCR, detection of PCR products by hybridization to an array of DNA targets has been suggested. The concept has been successfully implemented for several systems, including the differentiation among members of the *Fusarium solani* species complex of pathogens of solanaceous plants [54] and identification of numerous *Pythium* spp. [55]. Detection by hybridization allows high-level multiplexing, but the specificity of hybridization is lower than the specificity of PCR or melting curve analysis; careful optimization of hybridization conditions is required to prevent the hybridization of DNA of a single pathogen with several targets. A powerful PCR-based diagnostic system with high-level multiplexing and quantitative detection has been developed by BioTrove [56]. The method requires a complex proprietary instrumentation, which seems to no longer be available since the acquisition of BioTrove by Life Technologies in 2009 and the acquisition of Life Technologies by Thermo Fisher in 2014.

Among the molecular sequences used in *Fusarium* taxonomy, *RPB2* and *TEF-1α* have been used most frequently [57,58]. The distinction between similar sequences by HRM relies on differences in GC content, amplicon length, and the sequence [44]. In order to maximize the specificity of HRM analysis, short and highly polymorphic regions are used as amplicons [45,49,50]. The sequences of *RPB2* and *TEF-1α* genes used in the molecular taxonomy of *Fusarium* are too long for HRM; therefore, segments of the genes flanked by an established primer on one end and a new primer on the other end were amplified (Table 1). The location of both primers in highly conserved regions reduces the chance that the assay may fail for new isolates because of the lack of primer binding.

The assay fulfilled the purpose of detecting and distinguishing all eight major *Fusarium* pathogens infecting maize ears in Germany. DNA from some minor pathogens or saprophytes might generate indistinguishable melting curves, causing false positive signals. Due to their low abundance, however, the impact of these false positives on decisions about crop protection is expected to be negligible. To assess the specificity of the assay, melting temperatures of s*RPB2* and s*TEF-1α* amplicons were determined for an additional 12 *Fusarium* species (Table S4). Only *F. fujikuroi* and *F. proliferatum* could not be differentiated from the target species. *F. fujikuroi* does not colonize maize. *F. proliferatum* infects maize in some growing areas [5,11,12,15], but recent studies reported the species to be at a low abundance on maize in Poland [14], and essentially missing from maize in Germany [50]. Because the melting curves of s*RPB2* and s*TEF-1α* cannot distinguish between *F. proliferatum* and *F. temperatum*, an additional amplicon would be needed for the extension of the assay to *F. proliferatum*. 

In addition to the identification of eight *Fusarium* species by HRM analysis of s*RPB2* and s*TEF-1α* amplicons, the assay proved suitable for the identification of dominant pathogens in DNA extracted from naturally infected maize ears and for the identification of fungal colonies without DNA extraction by HRM followed by colony PCR. This shows that the technique is sufficiently robust to be used in routine diagnosis. Two of the eight species could not be discriminated by HRM analysis of a single amplicon, but were reliably distinguished by the melting profiles of another amplicon. An extension of the assay to further *Fusarium* species may require the integration of further amplicons, which could originate from the same gene or from other genes. The relatively high level of multiplexing and the simplicity of HRM assays, which work with universal primers and consist of a single run on a real-time thermocycler without further sample processing, truly compensates for the need to integrate additional amplicons with growing numbers of target species. The costs of HRM assays are lower than the costs of multiplex PCR with species-specific primers, TaqMan probes, hybridization of PCR products to immobilized species-specific targets, or DNA sequencing, let alone advanced technologies such as BioTrove’s OpenArray.

Field samples are often infected with multiple pathogens. The fact that our HRM assay is based on PCR primers that amplify DNA from multiple *Fusarium* species leads to two potential problems. First, amplicons in DNA extracted from samples infected with multiple pathogens will compete for primers, nucleotides, and DNA polymerase. The amplification of abundant pathogens may thus suppress the amplification of minor pathogens, preventing their detection. For the use of the assay in crop production, this does not pose a problem, because plant protection focuses on major pathogens. The second issue is that co-amplification of multiple amplicons may lead to the formation of hybrids, which melt at lower temperatures than the parent molecules. The presence of hybrids in amplification products will complicate the HRM patterns. Whether, and to what extent these hybrids may interfere with the assignment of amplicons/curves to taxa has to be further investigated. In the analysis of 100 naturally infected maize ears reported here, melting curves of hybrid amplicons were not detected. We suggest that this can be accounted for by the unequal abundance of pathogens in ears with mixed infection. Extrapolating the frequency of detection of dominant pathogens (see Section 2.2) to mixed infections, 6% of ears were likely infected concomitantly with the two most dominant pathogens, *F. graminearum* and *F. verticillioides*. If the abundance of pathogens in the infected ears was unequal, the melting curve of the less abundant pathogen and the melting curves of hybrid amplicons likely escaped detection. This will not pose a problem when the assay is used to guide crop protection against major pathogens. 

In monitoring programs that include isolation of fungal strains, melting curve analysis of amplicons generated by colony PCR might be used to identify minor pathogens or resolve ambiguous results of melting curve analysis of samples with mixed infection. The advantage of this approach is that the same technique is used for both the original analysis and the follow-up analysis of problematic samples. If melting curves are used in a routine diagnostic pipeline, the products of colony PCR can simply be inserted into the pipeline to be analyzed with the next sample batch. 

## 4. Material and Methods

### 4.1. Reference Strains, Sample Collection, and DNA Extraction

We selected eight *Fusarium* species (*F. cerealis, F. culmorum, F. equiseti, F. graminearum, F. poae, F. temperatum, F. tricinctum,* and *F. verticillioides*) for identification via HRM analysis. For each species, a reference strain (Table S1) was cultivated on potato dextrose agar plates for 5 to 7 days at 25 °C in the dark. For the comparison of melting temperature of amplicons, strains of 12 additional *Fusarium* spp. (Table S4) were grown in the same way. Mycelium was carefully scrubbed from the surface of the plates and lyophilized. In addition to reference strains, 100 naturally *Fusarium*-infected ears were collected from silage and grain maize, harvested at 30 field sites (one to nine ears per site) in seven federal states across Germany in 2017 (Figure S2). Ears were crushed, lyophilized, and finely ground to 1 mm using an ultra-centrifugal mill (ZM 200, Retsch, Haan, Germany). DNA from 30 mg naturally infected maize ears, as well as lyophilized mycelium of the reference strains, was extracted using a cetyltrimethylammonium bromide (CTAB)-based protocol [37]. Quality and quantity of the extracted DNA were assessed on agarose gels (0.8% (w/v) in 1 × Tris-acetate-EDTA buffer) stained with ethidium bromide. Gel electrophoresis was carried out for 60 min at 4.6 V/cm.

### 4.2. Fungal Colony PCR

Reference strains of *Fusarium* were cultivated as described above (see Section 4.1.), and aerial mycelium was carefully scrubbed from the surface of the plates using sterilized toothpicks and placed into a 1.5 mL tube containing 100 μL double-distilled water (ddH_2_O). For *F. cerealis, F. culmorum, F. graminearum, F. equiseti, F. temperatum, F. tricinctum*, and *F. verticillioides*, the mixture was incubated at 100 °C for 10 min and subsequently centrifuged at 16,000 × *g* for 30 s to pellet the mycelium. The obtained supernatant was transferred into a new 1.5 mL tube and directly used for PCR. For *F. poae*, the mycelium was briefly (approximately 10 s) introduced to the ddH_2_O. The mycelium was largely removed using the toothpick and colony PCR was performed from the remaining ddH_2_O without any further processing.

### 4.3. Primer Design and Maximum Likelihood Tree Analysis

We selected the *RPB2* and *TEF-1α* region for HRM analysis. In order to design primers flanking short and variable subsections of *RPB2* and *TEF-1α*, we first amplified both regions for the eight reference strains of *Fusarium* (Table S1), as described by Lofgren et al. [59] for *RPB2* and O’Donnell et al. [60] for *TEF-1α*. PCR products were purified and sent to Macrogen Europe for Sanger sequencing (Macrogen Europe B.V., Amsterdam, the Netherlands). The results were evaluated with Chromas version 2.6.6 (Technelysium Pty Ltd, South Brisbane, Australia). Multiple sequence alignment was then performed using ClustalW [61] in MEGA version 7.0.26 [62]. Alignments were processed in T-Coffee version 11.00y [63] and ESPript version 3.0 [64] (Figure S1). Two new primers suitable for HRM analysis were designed based on multiple gene alignments using the sequences of our eight reference strains (Table 1). Primer binding sites were conserved among species. We hereinafter refer to the selected subsections as s*RPB2* and s*TEF-1α*. The amplicon length was 304 bp for s*RPB2* and 247 bp for s*TEF-1α* (Table 1). Finally, a maximum likelihood analysis was conducted for s*RPB2* (1000 bootstrap replications) and s*TEF-1α* (without bootstrapping due to low sample size (*n* = 3)) using MEGA 7.0.26.

### 4.4. HRM Analysis 

PCR conditions were optimized using gradients of annealing temperature and final MgCl_2_ concentration. Amplification was performed in a CFX384 Thermocycler (Biorad, Rüdigheim, Germany) in 384 well microplates (SARSTEDT AG & Co. KG, Nümbrecht, Germany) with a total reaction volume of 4 μL. Reaction mixtures were composed of 1 μL template DNA or ddH_2_O for negative controls and 3 μL of reaction mixture (reaction buffer (20 mM Tris-HCl, 10 mM (NH_4_)2SO_4_, 10 mM KCl, 2 mM MgSO_4_, 0.1% Triton^®^ X-100, pH 8.8 at 25 °C); 0.5 mM MgCl_2_, 200 µM of each deoxyribonucleoside triphosphate (Bioline, Luckenwalde, Germany), 0.3 µM of each forward and reverse primer (Table 1); 3.3-time diluted EvaGreen^®^ solution (Jena Bioscience, Jena, Germany); 1 mg/mL bovine serum albumin, and 0.03 U Taq DNA Polymerase (New England Biolabs, Beverly, MA, USA)). Template DNA of the reference strains was adjusted to 100 pg μL^−1^ in ddH_2_O using gel densitometry [31]. DNA from naturally infected maize ears was diluted 1:100 in ddH_2_O before use in PCR. For fungal colony PCR, DNA was obtained as described in Section 4.2. Thermocycling conditions were as follows: 95 °C for 2 min (initial denaturation), 30 cycles of 94 °C for 30 s, 57 °C for 30 s, and 68 °C for 30 s. Final elongation was performed at 68 °C for 5 min. Following this, samples were heated to 95 °C for 30 s and cooled to 55 °C for 60 s. For fungal colony PCR, 35 cycles were performed. Fluorescence data for HRM analysis was obtained by increasing the temperature step-wise from 65 to 95 °C at 0.1 °C for 5 s per step with continuous fluorescence measurement. Reference strains of *Fusarium* species and negative controls (ddH_2_O) were amplified in triplicate.

### 4.5. Fluorescence Data Processing and Taxonomic Assignment

Relative fluorescence unit (RFU) data were obtained from CFX Maestro™ Software (Bio-Rad CFX Maestro 1.1 version 4.1.2433.1219) Biorad, Rüdigheim, Germany and analyzed in the R environment (version 3.6.1) [65]. RFU data were normalized by scaling all RFU values of each sample between 0 and 1. The negative first derivative (-d(RFU)/d(T)) was calculated employing the “diffQ2”-function in the R-package “MBmca” (version 0.0.3-5) and plotted to obtain normalized melting curves. The melting curves of the reference strains were generated from the mean fluorescence of the three technical replicates. Difference curves were obtained by subtracting the melting curve data of each reference strain or environmental sample from the mean melting curve data of all reference strains. Taxonomic identification was performed manually by carefully comparing the difference curves of the environmental samples against the reference strains.

## 5. Conclusions

Analysis of high-resolution melting (HRM) curves for the identification of *Fusarium* pathogens in plant material is an attractive technique for routine diagnostics in plant protection because it is cost-efficient, does not require any post-thermocycle sample processing, and allows multiplexing.

## Figures and Tables

**Table 1:** Primers used in this study.

Name	Sequence (5‘–3‘)	Gene	Amplicon Length (bp)	Reference
RPB2-5F2	GGGGWGAYCAGAAGAAGGC	*RPB2*	1200	[59]
RPB2-7CR	CCCATRGCTTGYTTRCCCAT			
EF1αF	ATGGGTAAGGARGACAAGAC	*TEF-1α*	694	[60]
EF1αR	GGARGTACCAGTRATCATGTT			
RPB2-5R1s	TCAACVACTTCCATACCTC	s*RPB2* *	304 (with RPB2-5F2)	This study
TEF-1aFs2	CAATAGGAAGCCGCYGAG	s*TEF-1α* *	247 (with EF1αR)	This study

* Short and variable subsections of *RPB2* and *TEF-1α*, which were selected for high-resolution melting (HRM) curve analysis.

## Supplementary Materials

The following materials are available online at https://www.mdpi.com/2076-0817/9/4/270/s1: Figure S1: Multiple sequence alignment of s*RPB2* and s*TEF-1α*, Figure S2: Sampling sites of naturally *Fusarium*-infected maize ears across Germany, Table S1: Reference strains of *Fusarium spp.* used in this study, Table S2: Melting temperature and GC-content of s*RPB2* and s*TEF-1α* amplicons of reference strains, Table S3: Species identification in naturally infected maize ears, Table S4: Melting temperature of s*RPB2* and s*TEF-1α* amplicons of additional *Fusarium* species, Table S5 and Table S6: Target sequences checked for binding of newly designed primers.
