# Supplementary material for: High-Resolution Melting (HRM) Curve Assay for the Identification of Eight Fusarium Species Causing Ear Rot in Maize
Source: Pathogens. 2020 Apr 7;9(4):270. doi: 10.3390/pathogens9040270 (PMC7238191; doi:10.3390/pathogens9040270)
Supplement: Supplementary file 1 [file pathogens-09-00270-s001.pdf]

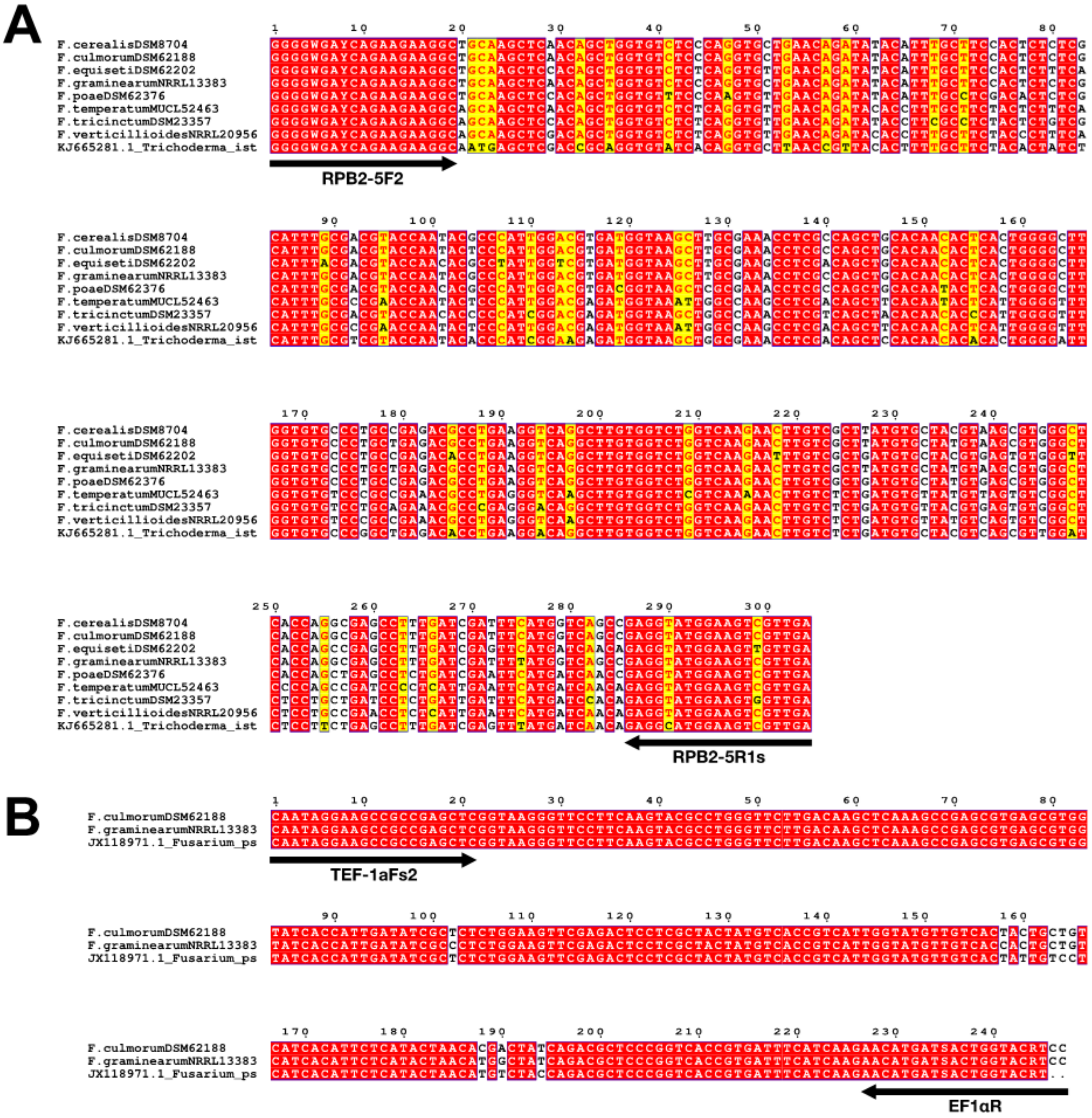

Figure S1. Multiple sequence alignment of s*RPB2* (**A**) and s*TEF-1α* (**B**). Analysis was performed with ClustalW [61] in MEGA version 7.0.26 [62]. Partial sequences of *RPB2* of *Trichoderma istrianum* (Accession KJ665281.1) and *TEF-1α* of *Fusarium pseudograminearum* (Accession JX118971.1) were included. Alignments were processed in T-Coffee version 11.00 [63] and ESPript version 3.0 [64]. Primers are shown as black arrows. *sRPB2* and *sTEF-1α* are short variable subsections of *RPB2* and *TEF-1α* selected for HRM analysis.

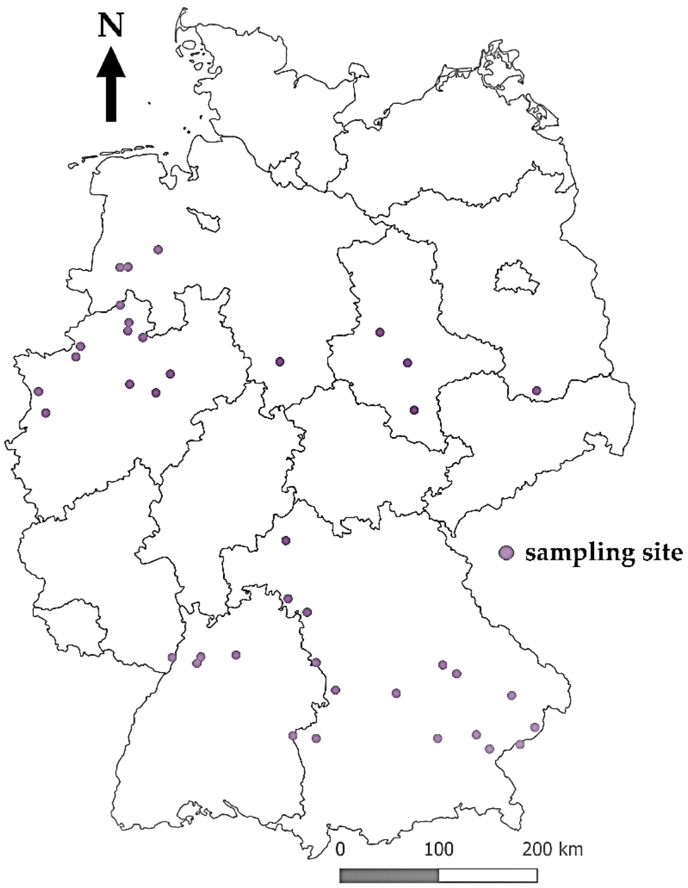

Figure S2. Sampling sites (n = 30) of the naturally *Fusarium*-infected maize ears across Germany. Black solid lines represent federal states. At each site, between 1 and 9 maize ears were collected. In total, 100 maize ears.

Table S1. Reference strains of *Fusarium* used in this study.

Strain ID[1]	Formae specials	Isolated from	Country of origin
BBA65106	*F. acuminatum*	Artemisia	Germany
BBA69055	*F. anguioides*	Soil	Japan
BBA71186	*F. arthrosporioides*	*Bellis*	Germany
DSM62161	*F. avenaceum* (Corda: Fr.) Saccardo	*Dianthus caryophyllus*	Germany
DSM8704	*F. cerealis* Cooke	Cereal debris	Australia
DSM62188	*F. culmorum* (W.G. Smith) Saccardo	*Zea mays*	Germany
DSM62202	*F. equiseti* (Corda) Saccardo	*Corylus avellana*	Germany
IMI58289	*F. fujikuroi*	*Oryza sativa*	Taiwan
NRRL13383	*F. graminearum* Schwabe	*Zea mays*	Iran
ESIER1400	*F. langsethiae*	*Triticum durum*	Italy
DSM62338	*F. oxysporum*	*Lycopersicon esculentum*	Italy
DSM62376	*F. poae* (Peck) Wollenweber	*Avena sativa*	Germany
DSM62261	*F. proliferatum*	*Cymbidium* hybrid	Germany
204.2c	*F. redolens*	*Zea mays*	Germany
DSM62423	*F. sporotrichioides*	*Pinus nigra*	Germany
CBS215.76	*F. subglutinans*	*Zea mays*	Germany
MUCL52463	*F. temperatum* Scauflaire J. & Munaut F.	*Zea mays*	Belgium
DSM23357	*F. tricinctum* (Corda) Saccardo	*Beta vulgaris*	Germany
RD15	*F. venenatum*	*Amaranthus retroflexus*	Germany
NRRL20956	*F. verticillioides* (Saccardo) Nirenberg	*Zea mays*	USA

[1]Fungal strains were obtained from German Collection of Microorganisms and Cell Cultures, Braunschweig, Germany (DSM); Westerdijk Fungal Biodiversity Institute, Baarn, Holland (CBS); Prof. Bettina Tudzynski, University of Muenster, Germany (IMI); Dr. Susanne Vogelgsang, Agroscope, Switzerland (ESIER); Prof. Tapani Yli-Mattila, University of Turku, Finland (BBA); and fungal collection of the laboratory (RD15, 204.2c).

Table S2. Melting temperature and GC-content of *sRPB2* and *sTEF-1α* amplicons of reference strains.

s***RPB2***		
***Fusarium* strain[1]**	**mean T_m ± SD[2]**	**GC-content (%)**
F. cerealis DSM8704	88.63 ± 0.06	54
F. culmorum DSM62188	87.90 ± 0.00[3]	53
F. equiseti DSM62202	87.97 ± 0.06[3]	51
F. graminearum NRRL13383	88.10 ± 0.00	53
F. poae DSM62376	89.00 ± 0.00	54
F. temperatum MUCL52463	86.27 ± 0.06	49
F. tricinctum DSM23357	87.50 ± 0.00	52
F. verticillioides NRRL20956	86.77 ± 0.06	50
s***TEF-1α***		
F. culmorum DSM62188	85.73 ± 0.06	49
F. graminearum NRRL13383	86.17 ± 0.06	50

[1] Fungal strains were obtained from German Collection of Microorganisms and Cell Cultures, Braunschweig, Germany (DSM); ARS culture collection, Peoria, IL, USA (NRRL); and Dr Jonathan Scauflaire, Earth and Life Institute, Louvain-la-Neuve, Belgium (MUCL). [2] Mean melting temperature (T_m) and standard deviation (SD) were calculated from three replicates. [3] s*RPB2* amplicons of *F. culmorum* and *F. equiseti* had the same melting temperature but could be distinguished with the help of an additional melting domain of the amplicon of F. *equiseti,* which caused a shoulder of the melting curve (Figure 2 C, E).

Table S3. Identification of dominant *Fusarium* species in naturally infected maize ears by HRM. Aliquots of DNA extracted from entire maize ears including rachis were used in the analysis.

***Fusarium* Species**	**Number of Ears**
F. graminearum	64
F. verticillioides	10
F. temperatum	6
F. poae	2
n.i.[1]	18

[1] *n.i.* = not identified.

Table S4. Melting temperature of s*RPB2* and s*TEF-1α* amplicons of additional *Fusarium* species.

sRPB2	
***Fusarium* species**[1]	**mean T_m**[2]
F. avenaceum DSM62161	86.90
F. proliferatum DSM62261	86.40
F. subglutinans CBS215.76	86.10
F. sporotrichioides DSM62423	88.40
F. fujikuroi IMI58289	86.40
F. oxysporum DSM62338	86.60
F. langsethiae ESIER1400	87.70
F. venenatum RD15	87.10
F. anguioides BBA69055	87.20
F. arthrosporioides BBA71186	87.00
F. acuminatum BBA65106	87.20
F. redolens 204.2c	86.20
sTEF-1α	
F. avenaceum DSM62161	- [3]
F. proliferatum DSM62261	86.00
F. subglutinans CBS215.76	85.70
F. sporotrichioides DSM62423	85.60
F. fujikuroi IMI58289	85.90
F. oxysporum DSM62338	85.90
F. langsethiae ESIER1400	85.40
F. venenatum RD15	86.10
F. anguioides BBA69055	86.00
F. arthrosporioides BBA71186	86.20
F. acuminatum BBA65106	85.60
F. redolens 204.2c	- [3]

[1] Fungal strains were obtained from German Collection of Microorganisms and Cell Cultures, Braunschweig, Germany (DSM); Westerdijk Fungal Biodiversity Institute, Baarn, Holland (CBS); Prof. Bettina Tudzynski, University of Muenster, Germany (IMI); Dr. Susanne Vogelgsang, Agroscope, Switzerland (ESIER); Prof. Tapani Yli-Mattila, University of Turku, Finland (BBA); and fungal collection of the laboratory (RD15, 204.2c). [2] Mean melting temperature (T_m) was calculated from two melting curves recorded with a temperature increment of 0.2 °C per step. [3] The PCR yield was not sufficient to record melting curves.

Table S5. Target sequences checked for binding of newly designed primers.

Fusarium Species	Primer[1]	Accession Number	Source[2]
F. cerealis	RPB2-5R1s	GQ915489.1	1
		MH582133.1	1
		ACZ55989.1	2
		GQ915489.1	2
	TEF-1aFs2	MF116382.1	1
		KP639706.1	1
		MH315942.1	1
		KX372274.1	1
F. culmorum	RPB2-5R1s	PTD11187.1	2
		GQ915490.1	1
		ACZ55991.1	2
		QBC75794.1	2
	TEF-1aFs2	MK952793.1	1
		MH315937.1	1
		MF511046.1	1
		MK034351.1	1
F. equiseti	RPB2-5R1s	MH582155.1	1
		MN692748.1	1
		QGR24120.1	2
		MN692734.1	2
	TEF-1aFs2	MK560312.1	1
		KU939021.1	1
		MG029527.1	1
		KU939015.1	1
F. graminearum	RPB2-5R1s	XM_011320308.1	1
		MH582206.1	1
		MN625697.1	2
		QGR24194.1	2
	TEF-1aFs2	MH315922.1	1
		MK560307.1	1
		MH572253.1	1
		MH122637.1	1
F. poae	RPB2-5R1s	GQ915495.1	1
		MH582128.1	1
		MG282392.1	1
		HQ646404.1	1
	TEF-1aFs2	KJ947343.1	1
		JF278599.1	1
		MH315912.1	1
		MG976797.1	1
F. temperatum	RPB2-5R1s	QBC75784.1	2
		QBC75507.1	2
		MH582217.1	1
		MH582076.1	1
	TEF-1aFs2	MH936000.1	1
		JX987069.1	1
		JX987071.1	1
		HM067687.1	1
F. tricinctum	RPB2-5R1s	MH582357.1	1
		HQ646398.1	1
		MH667554.1	2
		MH667555.1	2
	TEF-1aFs2	KM025422.1	1
		JX397856.1	1
		JX397845.1	1
		JX397871.1	1

F. verticillioides	RPB2-5R1s	XM_018898279.1	1
		MN193901.1	1
		MN193901.1	2
		QGX47694.1	2
	TEF-1aFs2	G550952.1	1
		KF715265.1	1
		KF499582.1	1
		KF499581.1	1

[1]Primer sequences are listed in Table 1. [2]Nucleotide sequences were obtained from [1] NCBI Genbank and from the [2] European Nucleotide Archive.

Table S6. Target sequences checked for binding of newly designed primers.

***Fusarium* Species**	**Primer[1]**	**Accession Number**	**Source[2]**
F. aethiopicum	RPB2-5R1s	AIX10119.1	2
		KM361670.1	1
	TEF-1aFs2	FJ240299.1	1
		FJ240295.1	1
F. asiaticum	RPB2-5R1s	MH582121.1	1
		MH582120.1	1
		JX171573.1	2
		QBC75592.1	2
	TEF-1aFs2	KT380123.1	1
		KT380118.1	1
		MK560334.1	1
F. austroamericanum	RPB2-5R1s	KM361661.1	1
	TEF-1aFs2	AF212439.1	1
		AF212438.1	1
		JF740836.1	1
		EF428587.1	1
F. boothii	RPB2-5R1s	GQ915487.1	1
		MH582075.1	1
		QBC75505.1	2
		ACZ55985.1	2
	TEF-1aFs2	MF133355.1	1
		KX881786.1	1
		MG588082.1	1
		MG588077.1	1
F. cortaderiae	RPB2-5R1s	AIX10103.1	2
		KM361662.1	1
	TEF-1aFs2	KR002048.1	1
		JQ740895.1	1
		EF428599.1	1
F. meridionale	RPB2-5R1s	KM361660.1	1
		AIX10099.1	2
	TEF-1aFs2	KY435748.1	1
		JF740835.1	1
		MN629330.1	1
F. mesoamericanum	RPB2-5R1s	KM361657.1	1
		AIX10093.1	2
	TEF-1aFs2	AF212442.1	1
		AF212441.1	1

[1]Primer sequences are listed in Table 1. [2]Nucleotide sequences were obtained from [1] NCBI Genbank and from the [2] European Nucleotide Archive.
